# Are Fermented Foods Effective against Inflammatory Diseases?

**DOI:** 10.3390/ijerph20032481

**Published:** 2023-01-30

**Authors:** Alok K. Paul, Chooi Ling Lim, Md. Aminul Islam Apu, Karma G. Dolma, Madhu Gupta, Maria de Lourdes Pereira, Polrat Wilairatana, Mohammed Rahmatullah, Christophe Wiart, Veeranoot Nissapatorn

**Affiliations:** 1 School of Pharmacy and Pharmacology, University of Tasmania, Hobart, TAS 7001, Australia; 2 Division of Applied Biomedical Science and Biotechnology, School of Health Sciences, International Medical University, Kuala Lumpur 57000, Malaysia; 3 Department of Nutrition and Hospitality Management, The University of Mississippi, Oxford, MS 38677, USA; 4 Department of Microbiology, Sikkim Manipal Institute of Medical Sciences, Sikkim Manipal University, Gangtok 737102, India; 5 Department of Pharmaceutics, School of Pharmaceutical Sciences, Delhi Pharmaceutical Sciences and Re-search University, New Delhi 110017, India; 6 CICECO-Aveiro Institute of Materials & Department of Medical Sciences, University of Aveiro, 3810-193 Aveiro, Portugal; 7 Department of Clinical Tropical Medicine, Faculty of Tropical Medicine, Mahidol University, Bangkok 10400, Thailand; 8 Department of Biotechnology & Genetic Engineering, University of Development Alternative, Lalmatia, Dhaka 1207, Bangladesh; 9 Department of Biomedical Sciences, Faculty of Medicine and Health Sciences, University Malaysia Sabah, Kota Kinabalu 88400, Malaysia; 10 School of Allied Health Sciences, World Union for Herbal Drug Discovery (WUHeDD), and Research Excellence Center for Innovation and Health Products (RECIHP), Walailak University, Nakhon Si Thammarat 80160, Thailand

**Keywords:** fermented food, fermented vegetables, inflammation, pain, arthritis

## Abstract

Fermented foods have been used over the centuries in various parts of the world. These foods are rich in nutrients and are produced naturally using various biological tools like bacteria and fungi. Fermentation of edible foods has been rooted in ancient cultures to keep food for preservation and storage for a long period of time with desired or enhanced nutritional values. Inflammatory diseases like rheumatoid arthritis, osteoarthritis, and chronic inflammatory pain are chronic disorders that are difficult to treat, and current treatments for these disorders fail due to various adverse effects of prescribed medications over a long period of time. Fermented foods containing probiotic bacteria and fungi can enhance the immune system, improve gastrointestinal health, and lower the risk of developing various inflammatory diseases. Foods prepared from vegetables by fermentation, like kimchi, sauerkraut, soy-based foods, or turmeric, lack proper clinical and translational experimental studies. The current review has focused on the effectiveness of various fermented foods or drinks used over centuries against inflammation, arthritis, and oxidative stress. We also described potential limitations on the efficacies or usages of these fermented products to provide an overarching picture of the research field.

## 1. Introduction

The chemical breakdown of complex biomolecules into simple molecules by microorganisms is referred to as fermentation. The term “fermented foods” refers to a set of foods that undergo various forms of chemical breakdowns induced by probiotic microorganisms [1]. Microorganisms consume vulnerable organic material as part of their metabolic activities, resulting in fermentation. Most fermented foods are made up of a complex mixture of carbohydrates, proteins, lipids, and other components that are transformed by various bacteria and enzymes simultaneously or sequentially. Several final products of the fermentation process, notably acids and alcohols, are antimicrobial [1]. During fermentation, bacteria or fungi make energy and extend their number in food ingredients. Compounds that have been totally degraded by fermentation to CO_2_ and water have lost all their energy values. Some health benefits of fermented foods containing probiotic organisms include enhancing the immune system, reducing lactose intolerance symptoms, improving intestinal tract health, and lowering the risk of certain cancers. Probiotics fight infections by modifying gut pH, producing antimicrobial chemicals, competing for pathogen binding and receptor sites as well as available nutrients and growth factors, activating immunomodulatory cells, and producing lactase, among other things. The fermenting organisms include *Leuconostoc*, *Streptococcus*, *Lactobacillus*, *Enterococcus*, *Aerococcus*, and *Pediococcus* spp., and several other fungi [2,3].

The fermentation of edible foods is steeped in ancient cultural practices as a natural, primitive, but effective, way of preservation. Using starter cultures such as lactic acid bacteria (LAB), the foods undergo significant biochemical changes, transforming raw materials into nutritious, flavorsome products that appeal to ethnic palates worldwide [4]. Yeast (*Saccharomyces cerevisiae*) and molds (*Aspergillus* sp., *Penicillium* sp.) are also commonly used to ferment alcoholic beverages and cheese and leaven bread [5].

In Asia, fermented foods typically involve non-dairy products such as fruits, vegetables, fish, and soybeans. Fermented vegetables and soy products take center stage as ‘superfoods’ as communities observe their health benefits beyond the nutritional value of pre-processed materials. Biologically active peptides derived from fermented products, including the anti-hypertensive conjugated linoleic acids (CLA), antimicrobial bacteriocins, and anti-carcinogenic sphingolipids, have since been discovered [6]. The functional anti-inflammatory and immunomodulatory properties of fermented plant foods owe their activity to the probiotic modulation of gut microbiota and the prebiotic effect from bioactive polyphenols generated by the fermentation process itself [7,8,9]. For instance, a mixture of 18 prebiotic vegetables inoculated with *Lactobacillus plantarum* (*Lactiplantibacillus plantarum*) was subjected to metabolomic analysis, and results showed a significant increase in levels of antioxidative and anti-inflammatory bioactive molecules such as lactate, 3-phennyllactate, and indole-3-lactate [10].

There is compelling experimental evidence that fermented vegetables or their derivatives, including preserved cabbage such as kimchi and sauerkraut, and soy-based fermented foods may potentially treat or prevent various inflammatory diseases [11]; however, clinical studies for many of these foods remain scarce. This section reviews the literature supporting the promising anti-inflammatory mechanisms of selected fermented vegetables while delineating the limitations of application in clinical medicine.

## 2. Kombucha 

Kombucha probably originated from Manchuria and is made by fermentation by a consortium of several symbiotic bacteria and yeasts [12]. It is a sweet green or black tea made by fermented cultures of symbiotic microorganisms like osmophilic yeast, *Acetobacter* bacteria (and *Lactobacillus* sp.) over a period of 10–14 days [13,14,15]. A recent report showed that Kombucha ready for consumption is composed of bacteria such as *Acetobacter musti* and *Gluconobacter potus*, and yeasts, namely *Dekkera bruxelensis*, *Schizosaccharomyces pombes*, *Hanseniaspora valbyensis, Brettanomyces anamalus*, *Pichia kudriavzevii*, *Starmerella vitis*, and *Saccharomyces cerevisiae* [16]. A recent review showed that Kombucha possesses antimicrobial properties toward a broad spectrum of bacteria and fungi as its microbiota composition may lead to producing acetic acid and various polyphenols [17]. Kombucha microbiota and tea can be used for fermented milk products that have higher food values [18]. Kombucha tea possesses anticancer, antimicrobial, and hepatoprotective properties [19]. Noticeably, the chemical composition of Kombucha depends on the ingredients used during its fermentation process, such as white-, green-, black-, and red-tea-derived Kombucha showed various levels (0.42–0.93 mg/L) of fluoride content; white or red tea produced the lowest amount of fluoride that is safe to eat [20]. Live bacteria and other organisms, including yeast are naturally present in Kombucha. The presence of flavonoids and other polyphenols in Kombucha inhibits oxidative enzymes and thus exerts anti-inflammatory effects [21]. The high antioxidant content of Kombuchas derived from oak is mostly related to their phenolic content and their capacity to reduce the production of nitric oxide, TNF-alpha, and IL-6 by lipopolysaccharides, demonstrating a significant anti-inflammatory action [22]. Kombucha consumption also reduced inflammation by increasing polarization of CD4+ T cells (by induction of IL-4 and TGF-β) and by inhibiting IFN-γ and IL-17 in a study on multiple sclerosis (an inflammatory disease) in an experimental autoimmune encephalomyelitis (EAE) in C57BL/6 mice [23]. Another study showed Kombucha prevented cellular immune function disorder at an early stage of sepsis in mice. Kombucha intake also promoted the growth of butyrate-producing bacteria in the gut that exert anti-inflammatory effects [24].

## 3. Fermented Turmeric

*Curcuma longa*, often known as turmeric, is a member of the Zingiberaceae family and is widely farmed in India, China, and other Southeast Asian countries [25]. *C. longa* is safe for human use as food, and it has long been used in Chinese culture and Ayurvedic medicine as an anti-inflammatory medication [26]. Turmeric has a yellow-pigmented component, which is mostly made up of curcuminoids. Curcumin, the main component of curcuminoids, is an antioxidant and anti-inflammatory compound that has been shown to help with osteoarthritis, type 2 diabetes, and dyslipidemia [27,28]. Numerous studies have demonstrated pharmacological properties and benefits of turmeric, including anti-Alzheimer activity [29,30], a hypolipidemic effect [31,32], anti-mutagenic activity [33], and antiprotozoal effects [34]. These are all related to the active ingredient curcumin [25]. The usefulness of turmeric extract (approximately 1000 mg/day of curcumin) in the treatment of arthritis is supported by scientific research [27]. Even though arthritis is linked to inflammation and pain, the actual origin of arthritis is multifactorial, and there is no absolute cure for the underlying reasons. The primary purpose of arthritis therapy is to alleviate joint discomfort caused by inflammation, regular wear and tear, and muscle strains [35]. Analgesics, steroids, and nonsteroidal anti-inflammatory medications (NSAIDs) are now used to treat arthritis. They alleviate symptoms such as extreme pain and inflammation [36]. NSAIDs are cyclooxygenase (COX) inhibitors that reduce inflammation by inhibiting prostaglandin and thromboxane production [36]. New NSAIDs block COX-2 specifically and are usually specific to inflammatory cells, lowering the incidence of peptic ulcer [27]. However, because of insufficient pain alleviation, immunological abnormalities, and dangerous gastrointestinal and cardiovascular side effects, their long-term usage is not recommended [37]. As a result, herbal remedies with anti-inflammatory characteristics and few side effects are recommended for the treatment of arthritis, particularly rheumatoid arthritis and osteoarthritis, particularly since several FDA-approved anti-inflammatory medicines have been withdrawn. The main bioactive element in turmeric is curcumin (diferuloylmethane). In *E. coli* lipopolysaccharide (LPS)-induced human monocytic macrophages and L929 fibroblasts, curcumin has been shown to interact with a variety of molecular targets in inflammation, including inhibiting the production of pro-inflammatory cytokines, tumor necrosis factor-alpha (TNF-α), and interleukin-1 (IL-1) [25]. It also stopped the production of nitric oxide and the activation of nuclear factor-kappa B (NF-kB), a gene that controls inflammation [38,39]. However, curcumin is a lipophilic polyphenol that is nearly insoluble in water, reducing its systemic bioavailability. Other studies have shown that, although curcumin has a limited impact on arthritis advancement in the Wistar rat model, microencapsulated curcumin successfully prevents arthritis progression, with the illness stabilizing after 10 days of treatment. It also lowered the amounts of immune cells (neutrophils and leukocytes), as well as pro-inflammatory cytokines such as TNF-α, IL-1, and IL-6, bringing them closer to the levels seen in arthritis-free people. Curcumin, in other forms, exhibited a lesser or no impact on arthritis progression [40]. Depending on the mode of administration, the same doses of curcumin exhibited a clearly stated beneficial anti-inflammatory impact. The microencapsulated curcumin showed the greatest promise for therapy. Microbial fermentation has been used to improve curcumin levels and pharmacological effects, as curcumin only makes up about 2–5% of turmeric [25]. The effects of turmeric fermentation using several types of lactic acid bacteria on its curcumin content and anti-inflammatory efficacy were investigated. Regardless of the quantity of *L. fermentum*-fermented turmeric used, fermentation with *Lactobacillus fermentum* (*Limosilactobacillus fermentum*) boosted curcumin concentration by 9.76% while causing modest cytotoxicity in RAW 246.7 cells [25]. The anti-inflammatory effect of fermented turmeric is mediated by a reduction in the c-Jun N-terminal kinase signal pathway, but not in unfermented turmeric, according to Western blot studies [25]. Although fermentation has boosted the curcumin content of turmeric, it is vital to validate the cytotoxicity of Lactobacillus-fermented turmeric. According to previous studies, the toxicity of unfermented turmeric is proportional to its concentration, with a high concentration causing a substantial amount of reactive oxygen species (ROS) in the cells and cell death [41,42]. The outcomes of this study corroborated prior studies, demonstrating that a large amount of unfermented turmeric reduced the viability of RAW 264.7 cells [25]. These findings revealed that fermenting turmeric by LAB improves curcumin content while also enhancing pharmacological action against arthritis.

A study on immunosuppressed rats with fermented turmeric (in camel milk) produced better immune biomarkers, increased anti-inflammation responses, and antioxidant activity compared with unfermented turmeric (in camel milk) supplementation [43]. Similarly, compared to non-fermented turmeric and turmeric that had been fermented by other probiotic strains, turmeric that had been fermented by *L. johnsonii* IDCC 9203 more effectively suppressed the production of the pro-inflammatory cytokines that were caused by lipopolysaccharide [44]. Another study shows antioxidant activity using the DPPH technique, and fermented turmeric demonstrated stronger antioxidative activity than raw turmeric. After 5 days of fermentation with *Bacillus natto*, fermented turmeric dramatically decreased the levels of aspartate aminotransferase (AST) and alanine aminotransferase (ALT) in contrast to unfermented turmeric. After fermentation, there was a considerable rise in HDL cholesterol and a significant decrease in LDL cholesterol [45].

## 4. Fermented Tea

Tea is one of the most consumed beverages in the world. Green tea, white tea, yellow tea, oolong tea, black (red) tea, and dark tea are the traditional classifications for tea based on the degree of fermentation. These teas come in a wide range of looks, infusion colors, tastes, and fragrances. Regardless, they all come from the same *Camellia sinensis* leaf [46]. Dark tea is the most thoroughly fermented tea. Dark tea is a fermented tea produced by solid-state fermentation with microorganisms, making it unique among these types [47]. Due to its distinct sensory properties, dark tea is becoming more popular among consumers. It is also gaining popularity because of its many health benefits, including protection against hypertension and cardiovascular disease, RA, weight reduction, metabolic syndrome alleviation, intestinal management, and changes in gut flora [48,49,50]. Microbial fermentation is assumed to be responsible for sensory properties of the dark tea as well as its health advantages. As a consequence of changes in chemical contents during microbial fermentation, several unique components associated with dark tea quality, such as catechin derivatives, flavonols, flavones, and their glycosides, phenolic acids, alkaloids, and terpenoids, have been discovered in recent years [51,52,53]. Degradation, oxidation, condensation, structural modification, methylation, and glycosylation have all been associated with changes in chemical components in dark tea [54,55,56]. Functional core microorganisms from the genera *Aspergillus*, *Eurotium*, *Candida, Bacillus*, *Pseudomonas*, and *Brevibacterium*, among others, are associated with these functions [47,48]. A variety of microbial metabolic and enzymatic processes have been discovered. The particular events that occur throughout the fermentation process are, however, still unknown. The color, taste, flavor, and scent of dark tea are due to oxidized polyphenolic chemicals such as theaflavins (TF) and thearubigins (TR) generated during fermentation [57]. The antioxidant properties of dark tea are due to the polyphenolic components TF, TR, and unoxidized catechins [58]. The antioxidant characteristics of these components are due to their capacity to scavenge free radicals, limit free radical production, and chelate transition metal ions [59]. Because of their propensity to form complexes with metals, TF, which are generated during fermentation and present solely in black tea, have an antioxidative impact. The conversion of catechins to TF during tea fermentation has no effect on the antioxidant properties of the tea [60,61]. However, no known mechanisms exist to explain the links between RA risk and tea use. Tea has anti-inflammatory as well as antioxidant effects [62]. In two distinct case-control investigations, antioxidants were shown to have a preventive effect against the development of RA [63,64,65].

## 5. Probiotics

**Figure 1:**
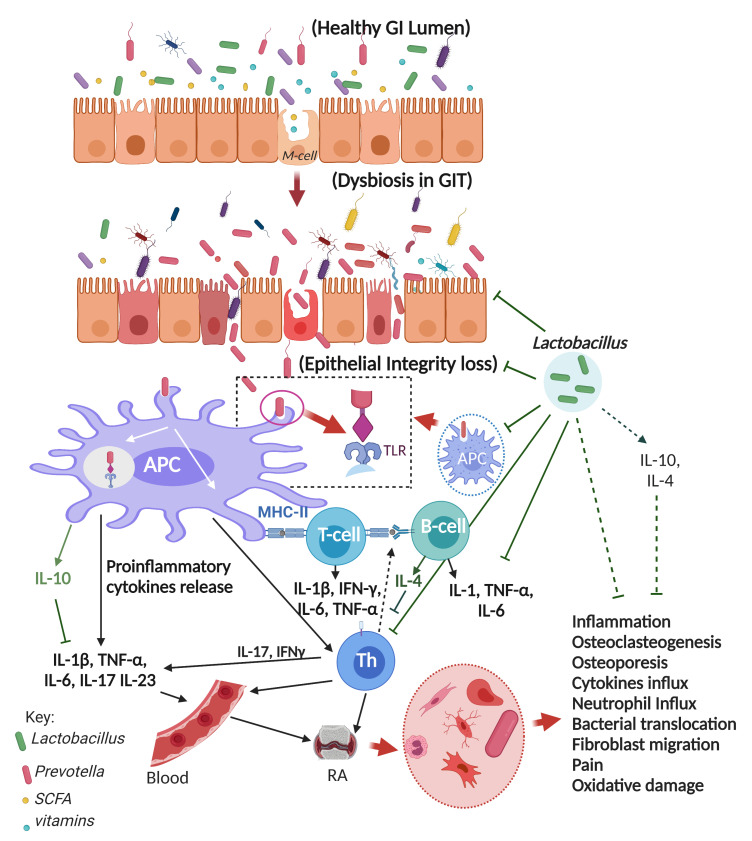
Gut dysbiosis and the role of probiotic bacteria (*Lactobacillus* spp.) against RA (adapted from [73]).

Microbes that encourage the development of other microorganisms were first designated as probiotics [66,67]. They’ve been more accurately characterized in recent years as mono- or mixed-cultures of live microorganisms that are advantageous to the host, increasing the qualities of the native microflora, whether supplied to an animal or a person. Probiotics are defined as food as preparations or nutritional supplements that promote human and animal health [66,67]. Their job is to get the human microflora back to its normal form after disruption due to poor diet, sickness, or the process of healing. The primary goal of ingestion, whether as food or as a medicine, is to improve the microbiota of the colon [68]. Probiotics are available in the form of capsules, pills, and tablets, as well as fermented foods [69]. To be evaluated for human usage, a probiotic strain must be isolated from human microflora, which gives it a high potential for adhesion to normal gastrointestinal cell walls. The variant should be safe for the host and not represent a hazard. *Lactobacillus, Pediococcus*, *Bifidobacterium*, *Lactococcus*, *Streptococcus,* and *Leuconostoc* are the probiotic bacteria most often isolated from fermented foods and animal/human digestive systems [70,71]. Many effects of probiotics, including the modulation of immunological systems, are dependent on the kind of probiotic. Certain strains have been demonstrated to boost the immune response, making them useful for immunocompromised individuals [72]. While the cause of RA is unknown, current evidence shows that bacterial dysbiosis at mucosal regions can contribute to the disease in physiologically vulnerable persons (Figure 1) [73]. Preclinical studies showed that *Lacticaseibacillus casei* or *L. acidophilus* over a period of 4 weeks prevented the development of arthritis by reducing inflammation (i.e., reduced proinflammatory cytokines) and oxidative stress. Similarly, intake of these *Lactobacillus* species caused increased levels of anti-inflammatory cytokines in the bloodstream [74]. Noticeably, oxidative stress reduction can also help to reduce RA and bacterial and viral inflammatory disorders [75]. Studies also show that the consumption of probiotic food or medications can help the prevention or provide symptomatic relief of dysentery, diarrhea, and various gastrointestinal disorders [76].

Autoantibodies related to serum in initial RA patients despite medically apparent synovitis support this notion, suggesting that illness originates outside the joint [77]. People with early-stage auto-immune provocative RA have a dysbiotic gut microbiota, which may provoke autoimmune responses in distant locations like the joints. Earlier mice models showed a relationship between gut microbiota and systemic immunity, as well as a link between joint provocative stimulation and systemic immunity. Prior probiotic studies failed to show a significant difference in RA activity when probiotics were used [78,79,80]. Recently, there has been a greater recognition based on diet for rheumatic illnesses. Eating a balanced diet and nutrient-enriched foods including fatty acids can introduce antioxidants that can reduce the risk of illness. [81] Similar results were found in a sample of 60 RA patients (30 cases and 30 controls). When compared to a placebo, researchers observed treatment with probiotics was connected to a significant improvement in insulin. Oxidative stress markers, however, showed no impact. In this example, 29 patients with RA took part in a single 12-week experiment, which indicated that combining *Lactobacillus rhamnosus* (*Lacticaseibacillus rhamnosus*) GR-1 and *L. reuteri* RC-14 resulted in a significant rise in the (HAQ) level in the probiotics groups but did not alleviate RA therapeutically [82,83]. Probiotic supplementation in RA patients has a general benefit, at least in the near term. Probiotic foods are a practical strategy to encourage the growth of beneficial bacteria in the stomach. Probiotics are bacteria and fungi that are alive; prebiotics like oligofructose and inulin provide nutrients that help the bacterial population in the gut to multiplicate and thus enhance the activities of probiotics. [83].

## 6. Kimchi

**Figure 2:**
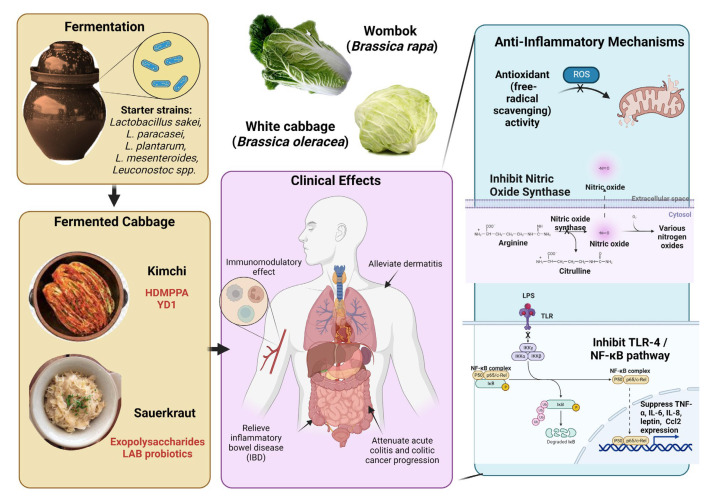
Preparation of Kimchi and its clinical effects. The figure was made with www.biorender.com (accessed on 10 September 2022).

One of the most well-recognized fermented vegetables commercially is kimchi—a traditional side dish consumed in Korea and popular across East Asia. Ancient Korean literature from 1145 A.D. (“Samkuksaki”) first detailed the dish as brine-fermented vegetables in stone jars, and since then many variations had evolved. In modern cuisine, kimchi consists mainly of Chinese cabbage (*Brassica rapa*) and radish fermented along with seasonings of red pepper, cinnamon, ginger, garlic, scallion, salt-pickled seafood, and soybean or fish sauce (jeotgal) (Figure 2) [8,84,85,86].

The purported health benefits of kimchi are numerous. Patra et al. highlighted the immense nutraceutical potential of kimchi as a functional food, encompassing antibacterial, antioxidative, cholesterol-lowering, immunomodulatory, and even neuroprotective properties [85,86,87,88,89]. Kimchi has also demonstrated potent radical scavenging and antioxidant activity in vitro, enhancing LLC-PK1 cell viability by protection against lipid peroxidation. Plausible mechanisms include the regulation of cyclooxygenase-2 (COX-2), inducible nitric oxide synthase (iNOS), and NF-κB signaling pathways [90]. A bioactive component in kimchi, 3-(4’-hydroxyl-3’,5’-dimethoxyphenyl) propionic acid (HDMPPA), was also found to alleviate levels of pro-inflammatory mediators and cytokines. Specifically, BV2 microglial cells stimulated with lipopolysaccharide (LPS) were ‘rescued’ from tumor necrosis factor-α (TNF-α), interleukin-1β, and NF-κB activation [91]. Recently, a novel antimicrobial peptide (YD1) isolated from kimchi was found to upregulate Nrf2 signaling and suppress NF-κB activation, subsequently depleting the levels of pro-inflammatory cytokines in both cellular and animal models [92]. 

The bioactivity against several inflammatory diseases may be contributed by the probiotic LAB derived from kimchi. Kwon et al. demonstrated evidence in a dermatitis (inflammatory skin condition) murine model, whereby the LAB strain *Lactobacillus sakei* WIKIM30 promoted regulatory T-cell (Treg) differentiation and modified gut microbiota populations [93]. Intestinal inflammatory conditions treated with microbes from kimchi were also well-studied, with the intragastric administration of *L. paracasei* (*Lacticaseibacillus paracasei*) LS2 curbing anorexia and promoting viability in dextran sulfate sodium-induced inflammatory bowel disease (IBD) mice [94]. *L. plantarum* (*Lactiplantibacillus plantarum*), another LAB strain from kimchi, suppressed pro-inflammatory gene TNF-α, IL-6, leptin, and Ccl2 expression in C57BL/6 mice on a high-fat diet (HFD) [95]. 

Similarly, the severity of acute colitis in C57BL/6 mice was attenuated by oral supplementation of *L. mesenteroides* and *L. sakei*, further cementing the positive anti-inflammatory effect of kimchi probiotics [96]. Non-lactobacillus kimchi starter strains such as *Latilactobacillus sakei* WIKIM31 and *Limosilactobacillus reuteri* EFEL6901 have been proven to enhance gut barrier functions and decrease nitric oxide production in LPS-induced macrophages, respectively [97,98]. In addition, the administration of fermented kimchi in a colitic cancer model attenuated interleukin inflammasomes, enhanced antioxidative agents, and conferred cytoprotection, whereas non-fermented kimchi did not [99].

## 7. Sauerkraut

Sauerkraut preparation dates back to the 4th century B.C. and is one of the most traditional methods of preserving cabbage. The nutritious fermented vegetable is more widely consumed in Central and Eastern Europe than Asian kimchi. Instead of mixing with spices and fish sauce, sauerkraut is produced from shredded fresh white cabbage (*Brassica oleracea* L. var) soaked in 2.0–3.0% sodium chloride, initiating the process of malolactic fermentation. Innately rich in lactic acid and vitamins, the popular dish was hailed as a health food and remedy for various ailments by ancient civilizations such as the Romans [8,100,101].

In a 2014 bibliometric analysis by Raak et al., out of 139 publications associating sauerkraut and human health, only 33 papers reported clinical effects such as anti-carcinogenicity [101]. More recently, a surge of interest in the health benefits of the fermented vegetable dish resulted in progressive evidence of its anti-inflammatory, antioxidant, and antimicrobial properties [8,11,102,103,104]. Probiotic LAB isolated from sauerkraut revealed that *Lactobacillus* spp. was the dominant genus, followed by *Leuconostoc* spp. (33%) [105], whereas another study isolated strains of Enterobacteriaceae and *Lactococcus* [106].

The anti-inflammatory properties of sauerkraut LAB were emphasized in a randomized, double-blinded pilot study on 34 Norwegian inflammatory bowel syndrome (IBS) patients. Supplementation with either the pasteurized or non-pasteurized dish for 6 weeks led to significant gut microbiota composition changes and alleviation of symptoms [102]. Using *Escherichia coli*-infected Balb-C mice, Zubaidah et al. revealed that sauerkraut fermented with *Leuconostoc mesenteroides* triggered immunomodulatory activity by enhancing both the adaptive and innate immune responses [107].

With the Northeastern Chinese sauerkraut (‘suan cai’), Xu et al. discovered exopolysaccharides that harbored radical scavenging and immunomodulatory activities via the assessment of nitric oxide, IL-6, TNF-α, and reactive oxygen species levels in RAW 264.7 macrophages [103]. This cousin of the European sauerkraut also had a naturally diverse microbial structure [104], which was reduced in richness through the inoculation of *Lactobacillus casei* (*Lacticaseibacillus casei*) 11MZ-5-1 starter cultures, thereby favorably leaving *Lactobacillus* strains in the predominant population [108]. In an in vitro model of intestinal barrier function, Huang and colleagues isolated *Weissella cibaria* MW01, a probiotic strain of LAB from Chinese sauerkraut. This strain showed a stronger adherence capacity compared to *L. rhamnosus* (*Lacticaseibacillus rhamnosus*) GG in the Caco-2 cell line, and significantly decreased LPS-induced inflammation through TNF-α, IL-6, and IL-8 attenuation [109].

Despite the availability of cellular and animal experimental models suggestive of health benefits from sauerkraut consumption, clinical data on its anti-inflammatory effects (aside from the Norwegian study [102]) is scarce and evidential value inadequate [100,110], warranting further investigation.

## 8. Soy-Based Fermented Foods

**Figure 3:**
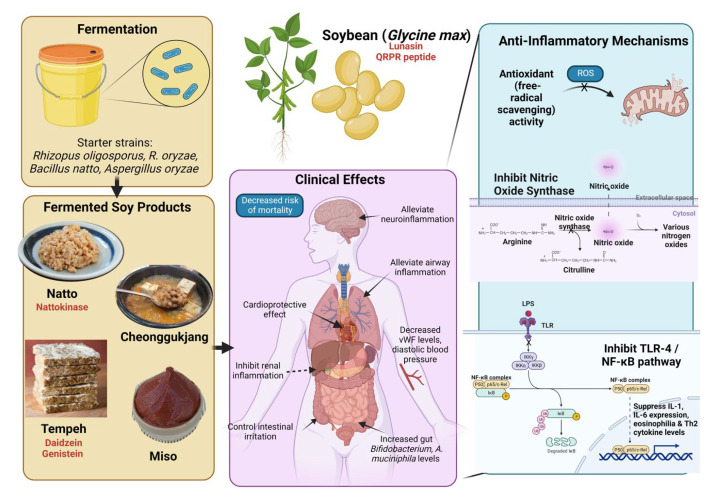
Anti-inflammatory effects of fermented soy-based products. The figure was made with www.biorender.com (accessed on 10 September 2022).

Food products from soy (*Glycine max*), a legume, are a staple in Asian households and form the basis for various high-protein beverages and dishes. A functional superfood, soybeans are packed with at least 14 bioactive phytochemicals encompassing phenolics, triterpenes, phytic acid, flavonoids, and carotenoids, in addition to isoflavones and dietary fibers [111] (Figure 3). A large body of evidence underscores the nutraceutical value of soy, ranging from anticancer, neuroprotective [112], anti-obesity, and conferring protection against renal, cardiovascular, and gastrointestinal pathology [111,113,114].

Soy products have sparked interest in recent years as an arsenal against inflammatory conditions. With the soy-derived anti-inflammatory peptide, lunasin, researchers perfected bioengineering methods to overexpress the protein, which suppressed nitric oxide, IL-1, and IL-6 levels [115]. Pan et al. showed in a RAW 264.7 cell inflammation model, the potential of the soybean peptide QRPR in suppressing interleukin levels and gene expression of inflammatory signaling pathways PIK3, AKT, and mTOR [113]. Using a rheumatoid arthritis (RA) albino rat model, El-Ashmawy et al. discovered that subcutaneous injection of isoflavone-free soy protein isolate (SPI) attenuated levels of RA markers, including anti-cyclic citrullinated peptide (anti-CCP) antibodies [116].

Clinically, a review of the association between inflammatory bowel diseases and diet found that supplementation with soy proteins modulated body fat composition, thus leading to the control of intestinal irritation [117]. In line with this, a recent meta-analysis of randomized control trials (RCTs) examined serum inflammatory markers in postmenopausal women showed evidence of a correlation between soy protein intake and reduced C-reactive protein levels [118].

In traditional Asiatic cuisine, soy is fermented and transformed into tempeh (popular in Indonesia), natto and miso (Japan), douchi (China), doenjang and cheonggukjang (Korea), and tofu (Asia). Instead of LAB, which is used in the production of sauerkraut and kimchi, the preparation of fermented soy foods generally begins with the inoculation of specific fungal strains or *Bacillus sp*. While tempeh is processed with *Rhizopus oligosporus* or *R. oryzae*, starter cultures for natto include *Bacillus natto*, and *Aspergillus oryzae* for both miso and douche [8,111,119,120]. Recent data from a prospective cohort study of 92,915 Japanese residents showed that higher consumption of fermented soy is linked to decreased risk of mortality compared to non-fermented soy [119].

Tempeh, a fermented soybean cake, is exceptionally known for its potent antioxidant capacity [120] (Figure 3). The product is rich in isoflavones daidzein and genistein (known as aglycones), which clearly inhibited free-radical scavenging and ferrous ion chelating activities [121], and these are influenced by the duration of fermentation [122], and microbial strain selection in starter cultures—*Bacillus* spp. and *Rhizopus* spp. [123]. Variations of the Indonesian tempeh have been developed to enhance antioxidant activity, including a gamma-aminobutyric acid (GABA)-rich product [124]. A pilot clinical study with a 28-day tempeh supplementation in 16 healthy women showed improved metabolic indicators and enhanced gut *Bifidobacterium* and *A. muciniphila* levels [125].

Neuroinflammatory conditions may lead to declining cognitive functions typical of Alzheimer’s, Parkinson’s, and other neurodegenerative diseases. In this respect, Korean fermented soy condiments doenjang, kanjang/ganjang, and cheonggukjang/chungkookjang have shown the potential to reverse or treat neuroinflammation. Studies suggest that free isoflavones produced during the fermentation of cooked soybean contribute to this capacity [126]. In an in vitro study of seven indole alkaloids from fermented Korean soy sauce (kanjang) administered to LPS-stimulated BV2 cells, two inhibited nitric oxide synthase, COX-2 expression, and the NF-κB pathway, highlighting the anti-neuroinflammatory action of the metabolites [127]. In addition, the poly-γ-glutamic acid (γ-PGA)-rich chungkookjang was found to prevent Alzheimer’s disease-induced memory impairment by modulating brain insulin sensitivity and interplay between the gut, microbiome, and brain [128]. Ko et al. demonstrated that a traditional fermented soybean paste, doenjang, can ameliorate neuroinflammatory features in C57BL/6J mice. Mice fed with doenjang-infused high-fat feed had reduced β-amyloid peptide (Aβ) and neuroinflammatory gene levels, further reinforcing the protective effect of fermented soy on the aging brain [129].

Several studies concur that fermented soy products may exert anti-inflammatory effects on various physiological systems. One such product contains nattokinase, a fibrinolytic enzyme isolated from natto, Japanese boiled soybeans fermented with *Bacillus subtilis*. Nattokinase (NK) is known to exhibit cardioprotective and hematological effects via its anti-inflammatory and antioxidant tendencies [130]. Jensen et al. (2019) reported in a randomized, double-blind, placebo-controlled clinical trial that 79 hypertensive subjects who consumed 100 mg NK per day for 8 weeks showed a decrease in von Willebrand factor (vWF) levels and diastolic blood pressure [131]. Research on RAW264.7 macrophages showed significant suppression of LPS-induced Toll-like receptor 4 (TLR4) and NOX-2 activation and downstream MAPK/NF-κB transcriptional pathways. Subsequently, NK-treated mice were also protected against LPS-induced acute renal trauma, concurring with cellular experiments [132]. In a massive, 15-year Japanese cohort study, natto intake was also correlated with decreased cardiovascular disease-associated deaths in adults of both genders [133].

Aside from naturally derived compounds from fermentation, ImmuBalance^TM^, a proprietary fermented soy product, alleviated airway inflammation in a murine asthmatic model through abrogation of eosinophilia and Th2 cytokine levels [134]. The administration of ImmuBalance^TM^ also significantly inhibited acute and chronic inflammation, features of chronic kidney disease pathogenesis, in female C57BL/6 mice. This was accompanied by decreased circulating kidney injury biomarkers [135].

## 9. Limitations and Adverse Effects

The very nature of vegetable fermentation, including diversity in production methods and starter culture strains, makes an objective assessment of its health benefits difficult. Additionally, despite burgeoning evidence favoring beneficial features, including the anti-inflammatory effects of fermented vegetables, there are underlying safety concerns regarding the frequent consumption of these functional foods.

One such concern is the high concentrations of biogenic amines such as tyramine and histamine. Sauerkraut has been found to harbor significantly elevated levels of tyramine, which counteracts monoamine oxidase inhibitor (MAOI) action in prescription drugs for mental health disorders, including depression and anxiety [101]. The relatively high histamine levels produced during the fermentation of sauerkraut may also be linked to an increased risk of allergic reactions, including hay fever and false-positive urticarial reactions in scratch tests. Nonetheless, the levels of biogenic amine by-products are strongly dependent on the storage duration, conditions, and strains of starter cultures [136].

The fermentation of kimchi requires copious amounts of salt, and this, in addition to increased nitrite and nitrate levels, may exert detrimental effects on human health if consumed in large quantities. This postulation is, however, countered by studies indicating that the salt and nitrate content in the dish, especially in modern preparations, may be negligible [85]. When kimchi fermentation is improperly done, pathogenic microbial contamination is a safety risk. There had been reports of food poisoning from aerobic bacteria, coliforms, *Escherichia coli*, *Bacillus cereus*, and even parasite eggs as hidden hazards. These are greatly reduced by thorough cleaning of the ingredients prior to fermentation [137]. Examination of 267 kimchi samples and 187 raw materials using the @RISK software showed that the probability of foodborne outbreaks in selected food service facilities was also minimal [138].

## 10. Conclusions

In summary, fermented vegetables such as kimchi, sauerkraut, fermented soy products, and beverages such as fermented teas are garnering attention as a source of natural anti-inflammatory bioactive compounds. These products should be considered functional foods as they provide both nutritional and therapeutic effects against various inflammatory chronic disorders. Nonetheless, there is a paucity of clinical and translational data, which highlights the need for thorough investigation using multicenter, randomized control clinical trials to systematically evaluate the potential effects of fermented foods.

## Data Availability

Not applicable.
